# Kinetic characterisation of arylamine *N*-acetyltransferase from *Pseudomonas aeruginosa*

**DOI:** 10.1186/1471-2091-8-3

**Published:** 2007-03-20

**Authors:** Isaac M Westwood, Edith Sim

**Affiliations:** 1 Department of Pharmacology, University of Oxford, Mansfield Road, Oxford, UK

## Abstract

**Background:**

Arylamine *N*-acetyltransferases (NATs) are important drug- and carcinogen-metabolising enzymes that catalyse the transfer of an acetyl group from a donor, such as acetyl coenzyme A, to an aromatic or heterocyclic amine, hydrazine, hydrazide or *N*-hydroxylamine acceptor substrate. NATs are found in eukaryotes and prokaryotes, and they may also have an endogenous function in addition to drug metabolism. For example, NAT from *Mycobacterium tuberculosis *has been proposed to have a role in cell wall lipid biosynthesis, and is therefore of interest as a potential drug target. To date there have been no studies investigating the kinetic mechanism of a bacterial NAT enzyme.

**Results:**

We have determined that NAT from *Pseudomonas aeruginosa*, which has been described as a model for NAT from *M. tuberculosis*, follows a Ping Pong Bi Bi kinetic mechanism. We also describe substrate inhibition by 5-aminosalicylic acid, in which the substrate binds both to the free form of the enzyme and the acetyl coenzyme A-enzyme complex in non-productive reaction pathways. The true kinetic parameters for the NAT-catalysed acetylation of 5-aminosalicylic acid with acetyl coenzyme A as the co-factor have been established, validating earlier approximations.

**Conclusion:**

This is the first reported study investigating the kinetic mechanism of a bacterial NAT enzyme. Additionally, the methods used herein can be applied to investigations of the interactions of NAT enzymes with new chemical entities which are NAT ligands. This is likely to be useful in the design of novel potential anti-tubercular agents.

## Background

Arylamine *N*-acetyltransferases (NATs, E.C. 2.3.1.5) are a family of enzymes (30–34 kDa) found in a range of eukaryotes and prokaryotes. NATs catalyse the transfer of an acetyl group from a donor, such as acetyl coenzyme A (AcCoA) to an aromatic or heterocyclic amine, hydrazine, hydrazide or *N*-hydroxylamine acceptor substrate.

The NAT enzymes in prokaryotes, particularly NAT from *S. typhimurium *[1], have been important in studies of the metabolism of carcinogens. Recent evidence suggests that prokaryotic NATs may also have an endogenous role. For example, a NAT-like protein in *Amycolatopsis mediterranei *(RifF) is responsible for the final ring-closure step in the biosynthesis of the rifamycin precursor, proansamycin X [2]. Although the precise endogenous function of NAT in mycobacteria has not been established, genetic studies suggest strongly that NAT has a role in cell wall complex lipid biosynthesis in *Mycobacterium bovis *BCG [3]. It has been proposed that NAT represents a good anti-tubercular target, since ablation of the *nat *gene results in increased intracellular killing of mycobacteria within macrophage [3]. The NAT from *Mycobacterium tuberculosis *has not been expressed as a soluble recombinant enzyme in sufficient quantities for detailed activity studies, although other bacterial NATs are available. These include NATs from *Salmonella typhimurium *[4], *Mycobacterium smegmatis *[5], *Mesorhizobium loti *[6] and *Pseudomonas aeruginosa *[7]. The three-dimensional structures of these enzymes have been solved by X-ray crystallography. The overall fold is superimposable [6-9], and all four enzymes share a catalytic triad of residues: Cys-His-Asp [9], a motif which is completely conserved throughout all known active NATs [10].

As a start to the process of identifying novel NAT ligands, Brooke and others have developed a method suitable for the rapid identification of NAT substrates and inhibitors with the colorimetric agent 5,5'-dithio-bis(2-nitrobenzoic acid) (Ellman's reagent, DTNB) [11]. The extent of reaction is measured by detecting the coloured 5-thio-2-nitrobenzoic acid, produced by reaction of DTNB with the free thiol CoA, formed during the NAT reaction [11,12]. Brooke and co-workers have used this method to identify novel inhibitors of bacterial NATs with the aim of developing novel antimycobacterial agents [13]. While the structures of bacterial NATs are known, to date, there have been no kinetic analyses of prokaryotic NAT enzymes and it is not known whether these enzymes share the kinetic mechanism previously determined for their eukaryotic counterparts.

The eukaryotic NAT enzymes have been investigated in relation to their role in drug- and carcinogen-metabolism [14-16], and it has also been suggested that certain eukaryotic NATs have an endogenous role [17]. The crystal structure of human NAT1 (F125S mutant, pdb code 2IJA) has recently been solved. This crystal structure, along with an NMR-derived model of human NAT1 [18] and homology models of human NAT1 [19], human NAT2 [20] and hamster NAT2 [21], all show that the three-dimensional fold is very highly conserved throughout the NAT family, with very similar positioning of the catalytic triad residues. The most notable difference between eukaryotic and prokaryotic NATs is the existence of a loop region between the second and third domains in eukaryotic NATs, which is unlikely to have a role in catalysis [21,22].

The determination of kinetic constants for NAT, in particular with AcCoA as the acetyl donor, is experimentally challenging [23,24]. The apparent Michaelis constants and limiting rates of *N*-acetylation are dependent on the concentrations of the acetyl donor and acceptor. There are several features of the reactants which limit the determination of kinetic parameters by linear methods, including limited solubility and very high optical absorbance. Despite the technical difficulties associated with NAT enzyme assays, the enzymatic reactions of NATs from pigeon liver [25] and rabbit liver [26] preparations have been shown to follow the Ping Pong Bi Bi kinetic mechanism [27]. Approximate kinetic parameters have been determined for several eukaryotic NAT enzymes [23,25,26,28-33], and Wagner, Hanna and colleagues have recently determined the catalytic mechanism of pure recombinant NAT2 from Syrian hamsters [34,35]. No such analysis has been carried out so far for any of the bacterial NATs.

The NAT enzyme from *P*. *aeruginosa *(PANAT) has been cloned, expressed, characterised and crystallised [7], and is an ideal enzyme system for the study of the kinetics of a bacterial member of this unique enzyme family. The PANAT enzyme is very stable and highly active relative to other bacterial NATs currently available as pure proteins [4,5,7].

## Results

### Determination of the kinetic mechanism and true kinetic parameters by normalised plot analysis

We have used a normalised plot method, described by Bravo and colleagues [36], to elucidate the kinetic mechanism of the PANAT-catalysed *N*-acetylation of 5-aminosalicylic acid with AcCoA as acetyl donor, and to measure the true kinetic parameters for the substrates.

A flowchart describing the normalised plot method is presented as supplemental data [see Additional file 1]. In this method, the normalised concentration (*A*') is defined as an arbitrary constant concentration (*A*) multiplied by a numerical factor (*a*) as shown in equation 1.

*A*' = *aA *    **(1)**

The initial rates of reaction are measured in three separate experimental series. For the series *b *= 1, reaction velocities (*V*_*a*,*b*_) are measured in the presence of a constant concentration of 5-aminosalicylic acid (*B*), and a series of actual concentrations of AcCoA which are described as multiples of the arbitrarily fixed concentration *A *(e.g. for a concentration of $\frac{A}{4}$, *a *= 0.25). Similarly, for the series *a *= 1, *V*_*a*,*b *_values are measured in the presence of AcCoA (at concentration *A*) and a series of concentrations of 5-aminosalicylic acid (multiples of *B*). For the final series of data (*a *= *b*), the concentrations of substrates are varied in identical proportions (e.g. $\frac{A}{4}$ and $\frac{B}{4}$, $\frac{A}{2}$ and $\frac{B}{2}$). The experiment is designed such that the initial reaction velocity where *a *= *b *= 1 (*V*_1,1_, eq. 2) is measured in each series of collected data. The normalised velocity, ${\bar{V}}_{a,b}$ (eq. 2), is a corrected reciprocal value of the experimentally determined value.

${\bar{V}}_{a,b}=\frac{V_{1,1}}{V_{a,b}}\;\left(2\right)$

The following experiments were performed with PANAT, AcCoA (A) and 5-aminosalicylic acid (B). The arbitrarily fixed values of *A *and *B *were 0.4 mM and 0.2 mM respectively. Assays were carried out in which the factors *a *and *b *were varied in the following series: *a *= *b *(0.25 to 15), *a *= 1 (*b *= 0.25 to 15) and *b *= 1 (*a *= 0.25 to 2), and the normalised velocities were calculated from the initial rates of reaction according to equation 2. Four possible rival kinetic mechanisms have been considered: Ping Pong Bi Bi with and without substrate inhibition, and Ordered Bi Bi with and without substrate inhibition. A normalised rate equation was derived (eq. 3) with three terms (*α*, *β *and *γ*, Table 1), which, when included in different combinations, allow these four possible rival kinetic models to be described (Table 2). The definitions of *α*, *β*, *γ *and *den *are given in Table 1.

**Table 1:** Definitions of parameters in the normalised velocity equation (eq 3)^a^

Parameter^*b*^	Definition^*c*^	Comments
*α*	*K*_iB_*K*_mA_	Appears in Ordered Bi Bi only
*β*	$\frac{K_{\text{mA}}b^{2}B^{2}}{K_{\text{si}(\text{B}\to \text{E})}}$	Accounts for substrate inhibition by 5-aminosalicylic acid on the free enzyme
*γ*	$\frac{aAb^{2}B^{2}}{K_{\text{si}(\text{B}\to_{\text{FP}}^{\text{EA}})}}$	Accounts for substrate inhibition by 5-aminosalicylic acid on the enzyme·AcCoA complex
*den*	$\alpha +K_{\text{mB}}A+K_{\text{mA}}B+AB+\frac{\beta }{b^{2}}+\frac{\gamma }{ab^{2}}$	The denominator term *den *is independent of both *a *and *b*

^*a *^The parameters *α*, *β *and *γ *are set to zero in different combinations to define different mechanisms.

^*b *^The definition of *γ *given here is for the Ping Pong Bi Bi mechanism (in which *α *= 0). *γ *for the Ordered Bi Bi mechanism is similar, however the denominator would be $K_{\text{si}(\text{B}\to_{\text{EPQ}}^{\text{EAB}})}$, relating to the binding of 5-aminosalicylic acid to either of the trimolecular EAB or EPQ complexes.

^*c *^The *K*_i _and *K*_si _terms are true dissociation constants for the enzyme·substrate complexes indicated, and *K*_m _values are Michaelis constants for the substrates indicated.

**Table 2:** Results of non-linear regression by the least-squares method.^a^

Mechanism^*b*^	1	2	3	4
Constant^*c*^	*α *= 0	*α *= *β *= 0		*β *= 0

*K*_mB_	0.219 ± 0.073**	0.199 ± 0.174^†^	0.219 ± 0.120^†^	0.199 ± 0.279^†^
*K*_mA_	1.02 ± 0.29**	1.51 ± 1.03^†^	1.02 ± 0.46*	1.51 ± 1.53^†^
*K*_si(B→E)_	1.93 ± 0.28***	-	1.93 ± 0.37***	-
$K_{\text{si}(\text{B}\to_{\text{FP}}^{\text{EA}})}$	1.03 ± 0.34**	0.550 ± 0.403^†^	1.03 ± 0.50^†^	0.550 ± 0.560^†^
*K*_iB_	-	-	~0.00 ± 0.01^†^	~0.00 ± 0.01^†^
*den*	0.422 ± 0.105***	0.512 ± 0.327^†^	0.422 ± 0.156*	0.512 ± 0.463^†^
*den*_(calc)_	0.408	0.491	0.408	0.491
Sum of squares	0.268	1.254	0.268	1.254
Bi Bi Mechanism	Ping Pong	Ping Pong	Ordered	Ordered
Substrate inhibition by 5-aminosalicylic acid on free enzyme?	Yes	No	Yes	No

^*a *^Least-squares non-linear regression was performed by using KyPlot [45].

^*b *^Mechanisms 1 and 2 refer to Ping Pong Bi Bi kinetics allowing 5-aminosalicylic acid to act as an inhibitor of the enzyme·AcCoA complex (EA). Mechanism 1 also accounts for 5-aminosalicylic acid binding to the free enzyme (E). Mechanisms 3 and 4 are directly analogous to 1 and 2 respectively, however they refer to Ordered Bi Bi kinetics.

^*c *^*K *values have dimensions of mM and *den *values are expressed in mM^2^. *α *and *β *are defined in Table 1. Variance and statistical significance values were determined within KyPlot by Student's t test. ^†^, P >= 0.05; *, P < 0.05; **, P < 0.01; ***, P < 0.001.

${\bar{V}}_{a,b}=\frac{\alpha +K_{\text{mB}}aA+K_{\text{mA}}bB+aAbB+\beta +\gamma }{ab\cdot den}\;\left(3\right)$

**Figure 1:**
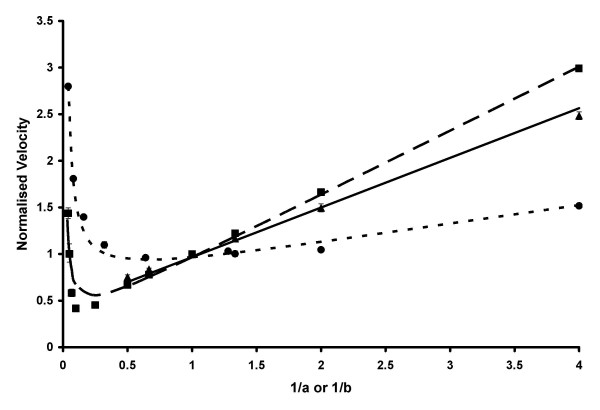
**Comparison of calculated and experimental kinetic data**. The calculated and experimental normalised data for the PANAT-catalysed acetylation with the substrates AcCoA (A) and 5-aminosalicylic acid (B) is shown. The points represent the experimental data in three series: *a *= *b *(■), *a *= 1 ●) and *b *= 1 (▲), expressed as the mean ± standard deviation of triplicate measurements. The lines represent values obtained through least-squares non-linear regression: *a *= *b *(long dashes), *a *= 1 (short dashes) and *b *= 1 (solid line). The values of the normalised substrate concentration constants *A *and *B *(eq. 1) were 0.4 and 0.2 mM respectively, and the normalised velocity is defined in equation 2. Both *x*- and *y*-coordinates are dimensionless. Reactions were performed in triplicate at 25°C and pH 8.0 as described in Methods.

The data shown in Figure 1 were then simultaneously fitted by least-squares non-linear regression to equation 3 a total of eight times, each with different combinations of the variables *α*, *β *and *γ *set to zero. When *γ *was set to 0, corresponding to no inhibition by 5-aminosalicylic acid on the enzyme·AcCoA complex (EA) complex, no solutions could be obtained through the non-linear regression analysis. The results of non-linear regression with the remaining four combinations, corresponding to four different mechanisms, are shown in Table 2. The mechanisms named 1P and 2P are Ping Pong mechanisms, and those named 3O and 4O are Ordered mechanisms. Substrate inhibition by 5-aminosalicylic acid on both the free enzyme (E) and the EA complex are accounted for by mechanisms 1P and 3O, and inhibition by 5-aminosalicylic acid on only EA is accounted for by mechanisms 2P and 4O. The self-consistency factor, *den*, was compared to the calculated value *den*_(calc) _by using the other constants obtained from the non-linear regression analysis. The similarity of *den *and *den*_(calc) _gives an indicator of the scientific reliability of the fit, while the sum of squares from least-squares non-linear regression indicates the mathematical accuracy of the fit [36].

The Ordered Bi Bi term *α *was found to reduce to zero when included in the calculation, hence the numbers generated with equations derived for mechanisms 3O and 4O were the same as those from mechanisms 1P and 2P respectively. In all cases, the *den *and *den*_(calc) _values were very similar; however, the sum of squares from the calculations where *β *was set to 0 were over four-fold higher than those where *β *was included. The statistical significance of each solution (determined with Student's t test within KyPlot), indicates that mechanism 1P is the most likely. In order to determine by independent means whether mechanism 1P or 2P is most appropriate, the method described by Mannervik was used [37], in which the quotient, *F*_exp _(eq. 4) is compared with the *F*-statistic: *F*(*p*_k _- *p*_j_, *n *- *p*_k_), where *SS*_j _(= 1.254) is the residual sum of squares from the simpler model (mechanism 2P) with *p*_j _(= 4) parameters, *SS*_k _(= 0.268) is the residual sum of squares from the more complex model (mechanism 1P) with *p*_k _(= 5) parameters and *n *(= 27) is the number of data points.

$F_{\exp}=\frac{\left(SS_{\text{j}}-SS_{\text{k}})(n-p_{\text{k}}\right)}{(p_{\text{k}}-p_{\text{j}})SS_{\text{k}}}\;\left(4\right)$

**Figure 2:**
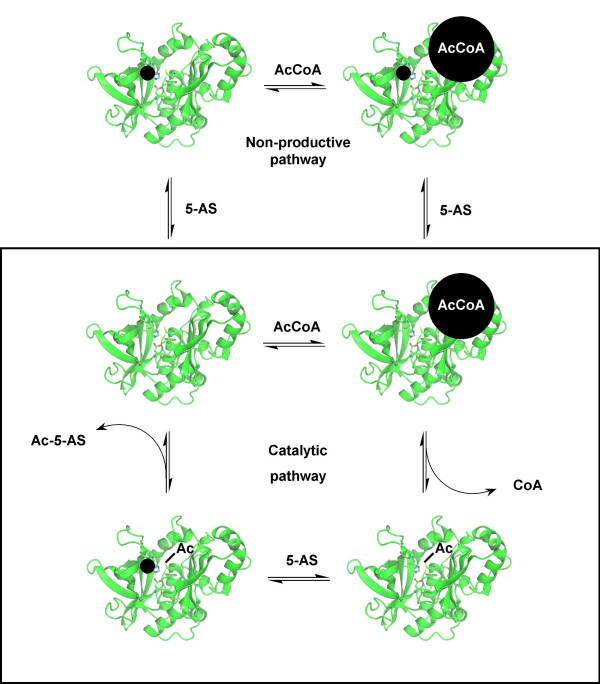
**The proposed kinetic mechanism of PANAT-catalysed *N*-acetylation of 5-aminosalicylic acid with AcCoA**. The schematic diagram shows the catalytic cycle (boxed) and the non-productive binding of 5-AS (●) to the free form of the enzyme and to the enzyme·AcCoA complex. It is not known whether AcCoA is able to bind to the enzyme·5-AS complex.

According to this method, the experimental quotient *F*_exp _was 80.9, and the critical value for *F*(1,22) is 14.38 at a value of *α *= 0.001: therefore, the reaction follows mechanism 1P, with substrate inhibition by 5-aminosalicylic acid on both the free form of the enzyme and the enzyme·AcCoA complex. A schematic diagram illustrating this mechanism is given in Figure 2. Therefore, for concentrations of AcCoA at 800 μM and below, the rate equation for this reaction is as shown in equation 5.

$v=\frac{V_{\max}aAbB}{K_{\text{mB}}aA+K_{\text{mA}}bB+aAbB+\frac{K_{\text{mA}}b^{2}B^{2}}{K_{\text{si}(\text{B}\to \text{E})}}+\frac{aAb^{2}B^{2}}{K_{\text{si}(\text{B}\to_{\text{FP}}^{\text{EA}})}}}\;\left(5\right)$

Thus, from the four rival kinetic mechanisms considered (Table 2), an Ordered mechanism can be ruled out, based on the reduction of the denominator term *α *to zero in the calculation process, the correlations of the rival kinetic models and the statistical significance of the fits. Of the two remaining Ping Pong mechanisms, mechanism 2P is a poorer fit than mechanism 1P, as shown clearly by the observation of substrate inhibition by 5-aminosalicylic acid, the correlation of calculated and experimental self-consistency factors (*den vs. den*_*calc*_) and the *F*-test comparisons. Therefore, the theoretical model which best fits the experimental data is a Ping Pong Bi Bi mechanism with substrate inhibition by the acceptor substrate 5-aminosalicylic acid on both the free form of the enzyme and the enzyme·AcCoA complex.

### Determination of the half-life of the acetylated enzyme intermediate

**Figure 3:**
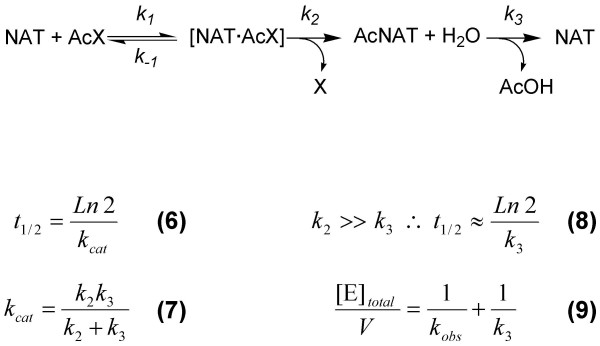
**The steady-state formation and hydrolysis of the acetyl-enzyme intermediate**. The acetyl donor is denoted AcX, where X is *p*-nitrophenol or CoA. AcNAT refers to the acetylated enzyme intermediate. For a derivation of equation 9, see [34].

In the absence of an acetyl acceptor, the proposed NAT reaction scheme is shown in Figure 3. The proposed scheme is based both on this study and on previously described work with a eukaryotic NAT [34]. Study of the first half of the Ping Pong Bi Bi reaction in this way allows for the determination of the half-life (*t*_1/2_) of the acetyl-NAT intermediate, which is a measure of its stability. In the proposed reaction scheme, *t*_1/2 _of the acetyl-NAT intermediate is dependent on both *k*_2 _and *k*_3 _(equations 6 and 7, Figure 3). If the rate of enzyme acetylation is very much faster than the rate of acetyl-enzyme hydrolysis (that is, if *k*_2 _is very much larger than *k*_3_), then *t*_1/2 _of the acetyl-NAT intermediate may be approximated by the following equation: $t_{1/2}\approx \frac{Ln2}{k_{3}}$ (equations 6–8, Figure 3).

**Figure 4:**
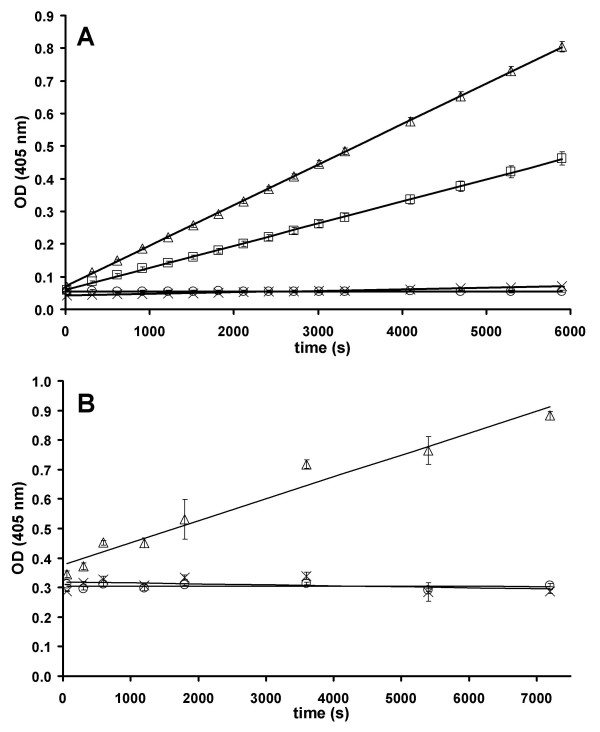
**PANAT-catalysed hydrolysis of acetyl donors**. PANAT at 8 μM (Δ), 4 μM (□) and a control using PBS buffer (×) were incubated with: **A**) *p*-nitrophenyl acetate (320 μM) or **B**) AcCoA (320 μM) at 25°C. The control experiments with PANAT (8 μM) and no acetyl donor (○) are also shown. Reactions were performed in quadruplicate at 25°C and pH 7.4 as described in Methods. The absorbance of *p*-nitrophenol or 5-thio-2-nitrobenzoic acid (produced by reaction with coenzyme A) is shown (*y*-axis) as a function of time (*x*-axis).

The rate of hydrolysis of the acetylated PANAT intermediate (AcNAT in Figure 3) has been determined under different experimental conditions where the acetyl donor is either *p*-nitrophenyl acetate or AcCoA, by measuring the rate of production of *p*-nitrophenol or CoA, and the results are shown in Figure 4. Equation 9 (Figure 3) was used to calculate the value of *k*_3 _for the reaction under each set of experimental conditions, from which the half-life of the acetyl enzyme intermediate was calculated. The data are summarised in Table 3.

**Table 3:** Determination of the half-life of the acetyl-enzyme intermediate.

Acetyl Donor	Rate of hydrolysis of acetyl donor (nM·s^-1^)^*a*^			*k*_3 _(× 10^-3^·s^-1^)^*b*^	*t*_1/2 _(s)^*c*^
			
	No enzyme	4 mM PANAT	8 mM PANAT		
*p*-Nitrophenyl acetate	0.28 ± 0.01	12.2 ± 0.5	22.8 ± 0.4	2.83 ± 0.08	235 ± 8
AcCoA	ND^*d*^	-	21.2 ± 3.9	2.66 ± 0.49	270 ± 49

^*a *^The rates of hydrolysis were determined spectrophotometrically by measuring the rate of production of *p*-nitrophenol or CoA, as described in the text.

^*b *^*k*_3 _values were determined according to equation 9 (Figure 3).

^*c *^*t*_1/2 _values were calculated according to equation 8 (Figure 3).

^*d *^ND, none detected.

The rates of enzyme-catalysed hydrolysis of *p*-nitrophenyl acetate were 12.2 ± 0.5 nM·s^-1 ^for 4 μM PANAT and 22.8 ± 0.4 nM·s^-1 ^for 8 μM PANAT after correction for the non-enzymatic rate of reaction, which was 0.28 ± 0.01 nM·s^-1^. These results give a *k*_3 _value of 2.83 ± 0.08 × 10^-3 ^s^-1^, which corresponds to a value for *t*_1/2 _of the acetyl-NAT intermediate of 235 ± 8 s.

The rate of enzyme-catalysed hydrolysis of AcCoA was 21.2 ± 3.9 nM·s^-1 ^for 8 μM PANAT. Under the experimental conditions used, no hydrolysis of AcCoA was observed in the absence of enzyme. The calculated *k*_3 _was 2.66 ± 0.49 × 10^-3 ^s^-1^, corresponding to a value for *t*_1/2 _of acetyl-PANAT of 270 ± 49 s. Thus, the half-life of the acetyl-PANAT intermediate is very similar when generated with *p*-nitrophenyl acetate or AcCoA as the acetyl donor.

### Comparison of AcCoA and *p*-nitrophenyl acetate as acetyl donors

The rates of acetylation of the acceptors: 5-aminosalicylic acid, 2-aminofluorene, hydralazine, *p*-aminobenzoic acid, *p*-anisidine, isoniazid and aniline (500 μM) by PANAT with 400 μM *p*-nitrophenyl acetate as acetyl donor were determined by measuring the formation of *p*-nitrophenol spectrophotometrically at 405 nm. The specific activities were calculated, after correcting for the non-enzymatic and enzyme-catalysed hydrolysis of *p*-nitrophenyl acetate, and are shown in Table 4. Solvent (DMSO) was found to have no effect on the rate of reaction at a final concentration of 5%. The experimentally determined specific activities for acetylation of acceptor substrates with the donor *p*-nitrophenyl acetate were compared with previously reported values where the acetyl donor was AcCoA (Table 4). For the substrates used in this study, the rates of acetylation with *p*-nitrophenyl acetate were between 1.1-fold and 71-fold slower than the corresponding rates with AcCoA as the acetyl donor (Table 4).

**Table 4:** Comparison of PANAT-catalysed *N*-acetylation with *p*-nitrophenyl acetate or AcCoA as acetyl donor^a^

Substrate	Specific Activity – PNPA (nmol·min^-1^·mg^-1^)^*b*^	Specific Activity – AcCoA (nmol·min^-1^·mg^-1^)^*c*^	Fold Difference
5-Aminosalicylic acid	1040 ± 30	73300 ± 3300	70.5
2-Aminofluorene	1470 ± 40	44710 ± 2720	30.4
Hydralazine	2990 ± 10	29550 ± 3110	9.9
*p*-Aminobenzoic acid	841 ± 17	8200 ± 78	9.8
*p*-Anisidine	2220 ± 40	13500 ± 0	6.1
Isoniazid	602 ± 3	2324 ± 0	3.9
Aniline	567 ± 7	629 ± 40	1.1

^*a *^The rate of production of *p*-nitrophenol was followed as described in Materials and Methods. Assay mixtures (100 μL) contained PANAT (50 ng), *p*-nitrophenyl acetate (400 μM) and acceptor substrate (500 μM) in PBS buffer with 5% (v/v) DMSO. Reactions were performed at 25°C, and specific activities are expressed as the mean ± standard deviation from triplicate measurements.

^*b *^PNPA, *p*-nitrophenyl acetate.

^*c *^Specific activities with AcCoA as acetyl donor are the literature values determined under similar experimental conditions [7].

When the acetyl donor *p*-nitrophenyl acetate is used, the product of the initial enzyme acetylation step is *p*-nitrophenol. The rate of the PANAT-catalysed acetylation of 5-aminosalicylic acid with either of the acetyl donors *p*-nitrophenyl acetate or AcCoA was not changed in the presence of *p*-nitrophenol at concentrations up to 100 μM. Therefore, it is unlikely that the slower rate of reaction with *p*-nitrophenyl acetate compared to AcCoA observed in the present study is due to product inhibition by the *p*-nitrophenol produced in the first half of the Ping Pong Bi Bi reaction. When AcCoA is used as the acetyl donor, CoA is the product of the initial enzyme-acetylation step. Andres and colleagues noted marked product inhibition by CoA [24], which may explain the reported results with the STNAT enzyme and NAT from pigeon livers, where the rates of acetylation of acceptor substrates were faster when *p*-nitrophenyl acetate was used instead of AcCoA as the acetyl donor [23,38]. Product inhibition by CoA does not explain the results obtained in this study with the PANAT enzyme; however, it is possible that the 71-fold slower acetylation of 5-aminosalicylic acid with *p*-nitrophenyl acetate as the acetyl donor instead of AcCoA may be due to the substrate inhibition exhibited by 5-aminosalicylic acid. As shown in this study, 5-aminosalicylic acid binds to the free form of the enzyme and the enzyme·AcCoA complex, both of which are inhibitory pathways in the proposed reaction mechanism (Figure 2). As 5-aminosalicylic acid is structurally more similar to *p*-nitrophenyl acetate than to AcCoA, it is possible that the effects of the observed substrate inhibition mechanisms would be greater when *p*-nitrophenyl acetate is used as the acetyl donor instead of AcCoA.

## Discussion

This study shows that NAT from *P. aeruginosa *follows the Ping Pong Bi Bi kinetic mechanism [27], in which the enzyme binds AcCoA, and the acetyl group is transferred to the enzyme. CoA is released, leaving a stable acetyl-enzyme intermediate, which then binds the second substrate. The acetyl group is transferred, regenerating the native enzyme and *N*-acetylated substrate. While various previous studies have shown that eukaryotic NAT enzymes follow Ping Pong Bi Bi kinetics [23,25,26], this study constitutes the first such analysis reported for a prokaryotic NAT. A summary of all kinetic parameters determined by the normalised plot method in this study is presented in Table 5. The normalised plot method allows for a full kinetic description of the system to be obtained with fewer required data points than conventional methods, and therefore reduced usage of expensive reagents, such as acetyl coenzyme A. The method also overcomes the experimental problems associated with the determination of apparent kinetic parameters, such as substrate inhibition. Structural studies and NMR experiments have previously demonstrated an interaction of the acetyl acceptor substrate, isoniazid, with NATs from *M. smegmatis *(MSNAT, [39]) and *S. typhimurium *(STNAT, [40]) in the absence of AcCoA. Sandy and colleagues suggested that the binding of isoniazid to MSNAT may constitute either a substrate inhibition complex or a pre-Ping Pong initiation step in the reaction mechanism [39]. The present study shows that the suggested pre-Ping Pong step is unlikely, and that binding of the acetyl acceptor substrate to the free form of the enzyme is an inhibitory pathway. We have shown that 5-aminosalicylic acid exhibits substrate inhibition by binding both to the free enzyme and to the enzyme·AcCoA complex. A schematic diagram of the proposed mechanism is presented in Figure 2. In the search for possible endogenous substrates and novel inhibitors of NAT enzymes by structure-based rational drug design, the enzyme·AcCoA complex and the acetyl-enzyme intermediate need to be studied as well as the native enzyme. A non-hydrolysable model of the acetyl-enzyme intermediate of MSNAT, in which the active-site cysteine residue is mutated to a glutamine, has been obtained by X-ray crystallography to a resolution of 1.45 Å [10]. Computational models of acetyl-enzyme·CoA and acetyl-enzyme·ligand complexes can therefore be generated by co-crystallisation studies with corresponding cysteine/glutamine mutants of other bacterial NATs.

**Table 5:** A summary of all kinetic constants determined for the PANAT-catalysed acetylation of 5-aminosalicylic acid

Constant^*a*^	Value
*K*_mA_	1.02 ± 0.29 mM
*K*_mB_	0.219 ± 0.073 mM
*K*_si(B→E)_	1.93 ± 0.28 mM
$K_{\text{si}(\text{B}\to_{\text{FP}}^{\text{EA}})}$	1.03 ± 0.34 mM
*V*_max_	1.75 ± 0.07 μM·s^-1^
*k*_cat_	434 ± 17 s^-1^
*k*_cat_/*K*_mA_	468 ± 150 mM^-1^·s^-1^
*k*_cat_/*K*_mB_	2259 ± 830 mM^-1^·s^-1^

^*a *^Abbreviations: A, AcCoA; B, 5-aminosalicylic acid; E, PANAT; F, acetyl-PANAT.

The first steps of any Ping Pong Bi Bi mechanism are the binding and reaction of the first substrate with the enzyme, resulting in the formation of a stable, non-native enzyme intermediate [27]. The isolation of a stable enzyme intermediate may be taken as positive evidence for a Ping Pong mechanism over an Ordered mechanism, in which all substrates bind to the enzyme prior to the release of the first product [27]. Figure 3 shows the generic reaction of NAT with an acetyl donor in the absence of acceptor substrate. The acetylation of hamster NAT2 with *p*-nitrophenyl acetate has been shown to be rapid, by stopped-flow techniques [34]. For hamster NAT2, the rate of hydrolysis of the acetyl-enzyme intermediate was found to be rate limiting, with a *k*_3 _of 7.85 ± 0.65 × 10^-3 ^s^-1 ^compared with an estimated *k*_2 _of 1301 ± 720 s^-1 ^and *k*_obs _of 44.8 s^-1 ^[34], which satisfies the assumptions that *k*_2 _>> *k*_3 _and *k*_obs _>> *k*_3 _(Figure 3). We have shown that PANAT catalyses the hydrolysis of both *p*-nitrophenyl acetate and AcCoA in the absence of acetyl acceptor substrate (Figure 4). Apart from two *C*-terminal truncation mutants of STNAT [38], no other bacterial NAT has been reported to exhibit this arylamine-independent activity. The lack of observed arylamine-independent acetyl-donor hydrolysis is most likely due to the significantly lower specific activity of the enzyme in the absence of an acetyl acceptor compared with when an acetyl acceptor is present [7], meaning that a high concentration of enzyme is required in order to measure the reaction rate of AcCoA hydrolysis in the absence of an acetyl acceptor. In the present study, the specific activity of PANAT-catalysed acetyl donor hydrolysis was 15,000-fold greater in the presence of the acceptor substrate 5-aminosalicylic acid compared with the acceptor-independent specific activity.

It has not been possible to detect an acetylated-STNAT enzyme intermediate by ^1^H and ^13^C NMR experiments [41], and it was concluded that, if an acetyl-STNAT intermediate were formed, its half-life would be considerably shorter than the timescale of the NMR experiments (~30 min). The half-life of acetyl-PANAT intermediate as determined in the present study (235 ± 8 s, Figure 4), is considerably shorter than the timescale of the NMR experiment. Jencks and colleagues measured the half-life of the acetylated NAT from pigeon liver as less than 1 min [32]. More recently, Wagner, Hanna and co-workers measured the half-life of the acetylated NAT2 enzyme from Syrian hamsters at 88.3 ± 8.3 s [34]. While the half-lives of the prokaryotic and eukaryotic enzymes are of a similar order of magnitude, the results obtained in this study suggest that the acetyl-PANAT intermediate may be more stable with respect to hydrolysis than both the acetyl-NAT intermediates from Syrian hamster and pigeon liver [32,34].

The apparent and true Michaelis constants for AcCoA from studies with pigeon liver NAT [29], hamster NAT2 [35], rabbit liver NAT [26], the full-length STNAT protein [1,38] and *C*-terminal truncation mutants of STNAT [38] are shown in Table 6. The true *K*_m _values for AcCoA appear to lie in the 1–6 mM range for the different enzymes, whilst the apparent Michaelis constants vary by approximately three orders of magnitude, depending on the enzyme and acetyl acceptor substrate used.

**Table 6:** Summary of Michaelis constants for AcCoA from different NATs.

Enzyme	True or Apparent *K*_m_	Acetyl Acceptor^*d*^	*K*_m_(mM)	Reference
PANAT	Apparent	*p*-anisidine (0.2 μM)	0.136	[7]
PANAT	Apparent	*p*-anisidine (2 mM)	0.466 ± 0.077	[see Additional file 2]
PANAT	True	5-AS	1.02 ± 0.29	Table 5
STNAT^a^	Apparent	*N*-OH-Glu-P-1	0.010	[1]
STNAT^a^	Apparent	INH	< 0.020	[38]
STNAT - 11^*b*^	Apparent	INH	0.393 ± 0.003	[38]
STNAT - 85^*c*^	Apparent	INH	0.764 ± 0.004	[38]
Pigeon liver NAT	Apparent	*p*-nitroaniline	0.007	[29]
Hamster NAT2	True	*p*-nitroaniline, pABA, pABA-Glu	5.94	[35]
Rabbit liver NAT	Apparent	INH	1.5	[26]

Selected Michaelis constants from the literature have been compiled along with those determined in this study for the purified PANAT enzyme.

^*a *^STNAT, NAT from *Salmonella typhimurium*.

^*b *^STNAT truncation mutant, missing 11 amino acids from the *C*-terminus.

^*c *^STNAT truncation mutant, missing the entire *C*-terminal domain (85 amino acids).

^*d *^Abbreviations: 5-AS, 5-aminosalicylic acid; *N*-OH-Glu-P-1, 2-hydroxyamino-6-methyldipyrido- [1,2-*a*:3',2'-*d*]-imidazole; INH, isoniazid; pABA, *p*-aminobenzoic acid; pABA-Glu, *p*-aminobenzoyl-L-glutamate.

The apparent kinetic parameters for AcCoA have been determined previously with recombinant PANAT enzyme and *p*-anisidine as the acetyl acceptor at a concentration of 200 μM [7]. In this study, we have used a higher concentration of *p*-anisidine (2 mM) to determine the apparent kinetic parameters for AcCoA under conditions that closer approach pseudo-first order kinetics [see Additional file 2]. The following values were obtained from a Hanes plot [see Additional file 2]: the apparent Michaelis constant for AcCoA (*K*_m,app_) of 466 ± 77 μM, apparent limiting rate (*V*_max,app_) of 362 ± 54 nM·s^-1^, apparent turnover number (*k*_cat,app_) of 17.5 ± 2.6 s^-1 ^and apparent specificity constant (*k*_cat,app_/*K*_m,app_) of 37.6 ± 5.4 mM^-1^·s^-1^. The previous studies with a lower concentration of *p*-anisidine as the acceptor substrate (200 μM) gave values for *K*_m,app _of 136 μM and *V*_max,app _of 153 nM·s^-1 ^[7]. At the relatively high acceptor substrate concentration of 2 mM, the apparent Michaelis constant was less than 50% of its true value (1.02 ± 0.29 mM), as determined with the normalised plot method in this study. Considering the very hydrophobic nature of many of the known acetyl acceptor substrates of NATs [42], it is not surprising that, in practice, the substrate concentrations required to reach pseudo-first order kinetics are often at or beyond the limit of solubility.

A summary of reported *K*_m _values for AcCoA with various NAT enzymes is given in Table 6. The very low apparent *K*_m _values for AcCoA with STNAT and NAT from pigeon livers may not be due solely to the experimental conditions used. For the reaction with hamster NAT2, it has been suggested that the deacetylation step with *p*-nitroaniline is rate limiting [35], which results in a decrease in the measured apparent *K*_m _for AcCoA compared with the true value. In the same study, the deacetylation step with *p*-aminobenzoic acid or *p*-aminobenzoyl-L-glutamate as the acetyl acceptor was found to be partially rate-limiting, thus the apparent Michaelis constants for AcCoA when using these substrates are also lower than the true value [35]. Therefore, it is likely that the comparatively low apparent *K*_m _values measured for STNAT [38] and pigeon NAT [23] reflect a rate-limiting deacetylation step in the presence of the acetyl acceptor substrates isoniazid, *N*-OH-Glu-P-1 and *p*-nitroaniline (Table 6).

The Michaelis constant for AcCoA with hamster NAT2 (5.94 mM) is nearly 6 times higher than the *K*_m _for AcCoA (1.02 ± 0.29 mM) determined with PANAT in the present study [35]. This indicates that AcCoA has a greater affinity for PANAT than for hamster NAT2. It is therefore possible that the longer half-life of the acetyl-PANAT intermediate compared to that of acetyl-NAT2 from hamsters may be due to the greater stability of the PANAT·AcCoA (or the acetyl-PANAT·CoA) complex.

**Figure 5:**
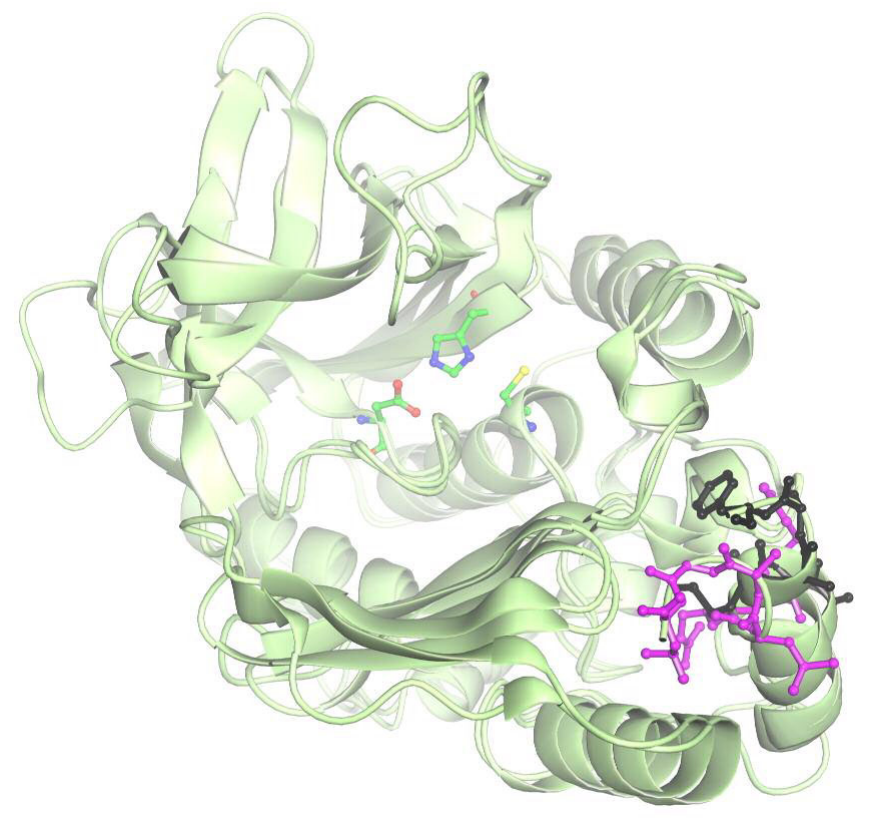
**A comparison of the *C*-terminal residues of STNAT and PANAT**. The active-site triad residues of PANAT (Cys-His-Asp) and the *C*-terminal residues of PANAT (purple) and STNAT (black) are shown in ball and stick representation. The distances between the active site Cys sulfur atom and residues Leu^276 ^and Phe^273 ^from PANAT and STNAT respectively are 25.8 Å and 17.6 Å respectively, and were determined by using SwissPDB Viewer [46]. The figure was produced with Aesop (M. E. M. Noble, unpublished results).

The *K*_m _value for AcCoA with PANAT is significantly larger than the *K*_m,app _value for AcCoA with the STNAT protein [38]. However, the two *C*-terminal truncation mutants of STNAT, reported by Mushtaq and colleagues [38], have apparent Michaelis constants which are very similar to the *K*_m _for AcCoA with PANAT. The STNAT sequence has eight more residues at the *C*-terminus than the PANAT sequence, and this *C*-terminal section of the STNAT is responsible for the observed differences in the truncation mutants compared with the full-length protein [38]. The comparatively shorter length of the *C*-terminus of PANAT may therefore be responsible for the higher apparent *K*_m _for AcCoA. The X-ray crystal structures of the two proteins are available, and a comparison of the *C*-terminal residues of these proteins indicates that a cleft which leads to the active site is blocked by the *C*-terminus of STNAT relative to that of PANAT (Figure 5). The terminal residue (Leu^276^) in the PANAT structure is 25.8 Å from the active site cysteine, compared to 17.6 Å between Phe^273 ^and the active site Cys in STNAT.

## Conclusion

We have shown that NAT from *Pseudomonas aeruginosa *follows a Ping Pong Bi Bi kinetic mechanism with substrate inhibition, by using a normalised plot method. This is the first reported study of the kinetic mechanism of a bacterial NAT. These studies provide a basis for the understanding of the molecular interactions of small-molecule ligands with NAT enzymes, and establish a reliable, reproducible and efficient method for the determination of kinetic parameters.

## Methods

### Protein preparation

Recombinant PANAT was prepared and purified as previously described [7]. Repeated concentration and dilution was used to exchange the buffer of protein solutions from the Tris.HCl buffers used during purification to phosphate-buffered saline (PBS, 0.137 mM NaCl, 2.7 mM KCl, 4.3 mM Na_2_HPO_4_, 1.4 mM KH_2_PO_4_, pH 7.4). Protein solutions were concentrated by using Amicon centrifugal filter devices (10,000 MW Cut-off, Millipore). The concentrations of solutions containing purified PANAT were determined spectrophotometrically with a Hitachi U2001 spectrophotometer by using the molar extinction coefficient (ε_280_) of 33120 M^-1^.cm^-1 ^(calculated with ProtParam [43,44]).

### Normalised plot analysis

The method of Bravo and colleagues [36] was used to determine the true kinetic parameters of the PANAT-catalysed acetylation of 5-aminosalicylic acid with AcCoA as the acetyl donor. A flowchart describing the approach is given as supplemental material [see Additional file 1]. The kinetic parameters are defined as follows: *K*_mA _and *K*_mB_, true Michaelis constants for substrates A (AcCoA) and B (5-aminosalicylic acid) respectively; *K*_iA_, dissociation constant of the EA complex (Ordered mechanism only); *K*_si(B→E)_, dissociation constant of the EB complex to free enzyme (E) and free substrate (B). The subscript 'si' refers to substrate inhibition by the substrate on the enzyme form indicated in brackets. The nomenclature proposed by Cleland [27] has been used throughout for enzyme forms and complexes; thus, E is the free form of PANAT and F is the acetyl-NAT intermediate. Non-linear regression analyses were performed by using KyPlot v2.0 beta 13 [45] with the least-squares method.

### Determination of acetyl-enzyme half-life

Assays measuring the hydrolysis of the acetyl donors *p*-nitrophenyl acetate and acetyl coenzyme A (AcCoA) were performed in the absence of an acetyl acceptor substrate. The assay solutions (100 μL) contained PANAT (8, 4 or 0 μM; 26.7, 13.3 or 0 μg) and either AcCoA or p-nitrophenyl acetate (320 μM) in PBS (pH 7.4) containing 5% (v/v) dimethylsulfoxide (DMSO). The reactions were started by the addition of acetyl donor (5 μL) in DMSO or PBS. The production of p-nitrophenol was followed spectrophotometrically at 405 nm in a continuous assay with a Hitachi U2001 spectrophotometer or a Tecan Sunrise 96-well plate reader; the molar extinction coefficient of p-nitrophenol at this wavelength (ε_405_) is 13400 M-1.cm-1, and data were collected at 30 s intervals. The rate of formation of CoA was determined spectrophotometrically (Tecan Sunrise 96-well plate reader) by following the reaction of CoA with the colorimetric agent 5,5'-dithio-bis(2-nitrobenzoic acid) (DTNB, 5 mM) in stop buffer (25 μL, 100 mM Tris.HCl, 6.4 M guanidine.HCl, pH 7.3) in a non-continuous assay as previously described [11,12]. All assays were performed at 25°C, and rates are relative to the non-enzymatic hydrolysis of AcCoA, expressed as the mean ± standard deviation of quadruplicate measurements.

### Determination of NAT-catalysed *N*-acetylation activity

Assays measuring the *N*-acetylation activity of PANAT were performed with the acetyl donors *p*-nitrophenyl acetate and AcCoA, and the acetyl acceptors 5-aminosalicylic acid, 2-aminofluorene, hydralazine, *p*-aminobenzoic acid, *p*-anisidine, isoniazid and aniline. Assay solutions (100 μL) contained PANAT (3.4 – 20.7 nM, 11 – 69 ng), acetyl donor (0.05 – 6.00 mM) and acetyl acceptor (0.05 – 3.00 mM) in 20 mM Tris.HCl buffer (pH 8.0) or PBS. Reactions were started by the addition of acetyl donor and the rates of production of *p*-nitrophenol or CoA were determined as described above. All assays were performed at 25°C, and controls were included in which no enzyme or no substrate was added. All rates are relative to the non-enzymatic hydrolysis of acetyl donor, and are expressed as the mean ± standard deviation of triplicate or quadruplicate measurements.

## Authors' contributions

IMW carried out the enzyme assays and kinetic analyses. ES conceived of the study. Both authors participated in the experimental design, the interpretation of the data and the drafting of the manuscript. Both authors confirm that they have read and approved the final manuscript.

## Supplementary Material

Additional file 1 Flow diagram describing the normalised plot method. A flow diagram outlining how data from the normalised plot method are analysed. Click here for file

Additional file 2 Hanes plot for AcCoA as a substrate of PANAT with the acetyl acceptor *p*-anisidine. This Hanes plot shows the determination of the apparent Michaelis constant for acetyl coenzyme A with the PANAT enzyme in the presence of the acceptor substrate *p*-anisidine. Click here for file

## References

[B1] Watanabe M Sofuni T Nohmi T Involvement of Cys69 residue in the catalytic mechanism of N-hydroxyarylamine O-acetyltransferase of Salmonella typhimurium. Sequence similarity at the amino acid level suggests a common catalytic mechanism of acetyltransferase for S. typhimurium and higher organisms. J Biol Chem 1992 267 8429 8436 1569093

[B2] Floss HG Yu TW Lessons from the rifamycin biosynthetic gene cluster Curr Opin Chem Biol 1999 3 592 597 10.1016/S1367-5931(99)00014-9 10508670

[B3] Bhakta S Besra GS Upton AM Parish T Sholto-Douglas-Vernon C Gibson KJC Knutton S Gordon S daSilva RP Anderton MC Sim E Arylamine N-acetyltransferase is required for synthesis of mycolic acids and complex lipids in Mycobacterium bovis BCG and represents a novel drug target J Exp Med 2004 199 1191 1199 10.1084/jem.20031956 15117974 PMC2211905

[B4] Sinclair JC Delgoda R Noble ME Jarmin S Goh NK Sim E Purification, characterization, and crystallization of an N-hydroxyarylamine O-acetyltransferase from Salmonella typhimurium Protein Expr Purif 1998 12 371 380 10.1006/prep.1997.0856 9535705

[B5] Payton M Auty R Delgoda R Everett M Sim E Cloning and characterization of arylamine N-acetyltransferase genes from Mycobacterium smegmatis and Mycobacterium tuberculosis: increased expression results in isoniazid resistance J Bacteriol 1999 181 1343 1347 9973365 10.1128/jb.181.4.1343-1347.1999 PMC93516

[B6] Holton SJ Dairou J Sandy J Rodrigues-Lima F Dupret JM Noble M Sim E Structure of Mesorhizobium loti arylamine N-acetyltransferase 1 Acta Crystallograph Sect F Struct Biol Cryst Commun 2005 61 14 16 10.1107/S1744309104030659 PMC1952398 16508079

[B7] Westwood IM Holton SJ Rodrigues-Lima F Dupret JM Bhakta S Noble ME Sim E Expression, purification, characterization and structure of Pseudomonas aeruginosa arylamine N-acetyltransferase Biochem J 2005 385 605 612 10.1042/BJ20041330 15447630 PMC1134735

[B8] Sandy J Mushtaq A Kawamura A Sinclair J Sim E Noble M The structure of arylamine N-acetyltransferase from Mycobacterium smegmatis - an enzyme which inactivates the anti-tubercular drug, isoniazid J Mol Biol 2002 318 1071 1083 10.1016/S0022-2836(02)00141-9 12054803

[B9] Sinclair JC Sandy J Delgoda R Sim E Noble ME Structure of arylamine N-acetyltransferase reveals a catalytic triad Nat Struct Biol 2000 7 560 564 10.1038/76783 10876241

[B10] Sandy J Mushtaq A Holton SJ Schartau P Noble ME Sim E Investigation of the catalytic triad of arylamine N-acetyltransferases: essential residues required for acetyl transfer to arylamines Biochem J 2005 390 115 123 10.1042/BJ20050277 15869465 PMC1184567

[B11] Brooke EW Davies SG Mulvaney AW Pompeo F Sim E Vickers RJ An approach to identifying novel substrates of bacterial arylamine N-acetyltransferases Bioorg Med Chem 2003 11 1227 1234 10.1016/S0968-0896(02)00642-9 12628650

[B12] Riddles PW Blakeley RL Zerner B Reassessment of Ellman's reagent Methods Enzymol 1983 91 49 60 6855597 10.1016/s0076-6879(83)91010-8

[B13] Brooke EW Davies SG Mulvaney AW Okada M Pompeo F Sim E Vickers RJ Westwood IM Synthesis and in vitro evaluation of novel small molecule inhibitors of bacterial arylamine N-acetyltransferases (NATs) Bioorg Med Chem Lett 2003 13 2527 2530 10.1016/S0960-894X(03)00484-0 12852958

[B14] Hein DW Molecular genetics and function of NAT1 and NAT2: role in aromatic amine metabolism and carcinogenesis Mutat Res 2002 506-507 65 77 12351146 10.1016/s0027-5107(02)00153-7

[B15] Kadlubar FF Badawi AF Genetic susceptibility and carcinogen-DNA adduct formation in human urinary bladder carcinogenesis Toxicol Lett 1995 82-83 627 632 10.1016/0378-4274(95)03507-9 8597119

[B16] Hanna PE N-acetyltransferases, O-acetyltransferases, and N,O-acetyltransferases: enzymology and bioactivation Adv Pharmacol 1994 27 401 430 8068562 10.1016/s1054-3589(08)61041-8

[B17] Minchin RF Acetylation of p-aminobenzoylglutamate, a folic acid catabolite, by recombinant human arylamine N-acetyltransferase and U937 cells Biochem J 1995 307 1 3 7717963 10.1042/bj3070001 PMC1136736

[B18] Zhang N Liu L Liu F Wagner CR Hanna PE Walters KJ NMR-based model reveals the structural determinants of mammalian arylamine N-acetyltransferase substrate specificity J Mol Biol 2006 363 188 200 10.1016/j.jmb.2006.08.026 16959263

[B19] Rodrigues-Lima F Delomenie C Goodfellow GH Grant DM Dupret JM Homology modelling and structural analysis of human arylamine N-acetyltransferase NAT1: evidence for the conservation of a cysteine protease catalytic domain and an active-site loop Biochem J 2001 356 327 334 10.1042/0264-6021:3560327 11368758 PMC1221842

[B20] Rodrigues-Lima F Dupret JM 3D model of human arylamine N-acetyltransferase 2: structural basis of the slow acetylator phenotype of the R64Q variant and analysis of the active-site loop Biochem Biophys Res Commun 2002 291 116 123 10.1006/bbrc.2002.6414 11829470

[B21] Kawamura A Graham J Mushtaq A Tsiftsoglou SA Vath GM Hanna PE Wagner CR Sim E Eukaryotic arylamine N-acetyltransferase. Investigation of substrate specificity by high-throughput screening Biochem Pharmacol 2005 69 347 359 10.1016/j.bcp.2004.09.014 15627487

[B22] Payton M Mushtaq A Yu TW Wu LJ Sinclair J Sim E Eubacterial arylamine N-acetyltransferases - identification and comparison of 18 members of the protein family with conserved active site cysteine, histidine and aspartate residues Microbiology 2001 147 1137 1147 11320117 10.1099/00221287-147-5-1137

[B23] Riddle B Jencks WP Acetyl-coenzyme A: arylamine N-acetyltransferase. Role of the acetyl-enzyme intermediate and the effects of substituents on the rate J Biol Chem 1971 246 3250 3258 5103348

[B24] Andres HH Klem AJ Szabo SM Weber WW New spectrophotometric and radiochemical assays for acetyl-CoA: arylamine N-acetyltransferase applicable to a variety of arylamines Anal Biochem 1985 145 367 375 10.1016/0003-2697(85)90376-8 4014668

[B25] Jenne JW Boyer PD Kinetic characteristics of the acetylation of isoniazid and p-aminosalicylic acid by a liver-enzyme preparation Biochim Biophys Acta 1962 65 121 127 10.1016/0006-3002(62)90155-5 13957595

[B26] Weber WW Cohen SN N-acetylation of drugs: isolation and properties of an N-acetyltransferase from rabbit liver Mol Pharmacol 1967 3 266 273 6037685

[B27] Cleland WW The kinetics of enzyme-catalyzed reactions with two or more substrates or products. I. Nomenclature and rate equations Biochim Biophys Acta 1963 67 104 137 10.1016/0006-3002(63)91800-6 14021667

[B28] Andres HH Klem AJ Schopfer LM Harrison JK Weber WW On the active site of liver acetyl-CoA. Arylamine N-acetyltransferase from rapid acetylator rabbits (III/J) J Biol Chem 1988 263 7521 7527 2897358

[B29] Andres HH Kolb HJ Schreiber RJ Weiss L Characterization of the active site, substrate specificity and kinetic properties of acetyl-CoA:arylamine N-acetyltransferase from pigeon liver Biochim Biophys Acta 1983 746 193 201 6882770 10.1016/0167-4838(83)90074-2

[B30] Andres HH Vogel RS Tarr GE Johnson L Weber WW Purification, physicochemical, and kinetic properties of liver acetyl-CoA:arylamine N-acetyltransferase from rapid acetylator rabbits Mol Pharmacol 1987 31 446 456 3574290

[B31] Jenne JW Partial purification and properties of the isoniazid transacetylase in human liver: its relationship to the acetylation of p-aminosalicylic acid J Clin Invest 1965 44 1992 2002 5851956 10.1172/JCI105306 PMC289702

[B32] Jencks WP Gresser M Valenzuela MS Huneeus FC Acetyl Coenzyme A : Arylamine Acetyltransferase (Measurement of the steady state concentration of the acetyl-enzyme intermediate) J Biol Chem 1972 247 3756 3760 5064220

[B33] Hickman D Palamanda JR Unadkat JD Sim E Enzyme kinetic properties of human recombinant arylamine N-acetyltransferase 2 allotypic variants expressed in Escherichia coli Biochem Pharmacol 1995 50 697 703 10.1016/0006-2952(95)00182-Y 7669073

[B34] Wang H Vath GM Gleason KJ Hanna PE Wagner CR Probing the mechanism of hamster arylamine N-acetyltransferase 2 acetylation by active site modification, site-directed mutagenesis, and pre-steady state and steady state kinetic studies Biochemistry 2004 43 8234 8246 10.1021/bi0497244 15209520

[B35] Wang H Liu L Hanna PE Wagner CR Catalytic mechanism of hamster arylamine N-acetyltransferase 2 Biochemistry 2005 44 11295 306 10.1021/bi047564q 16101314

[B36] Bravo IG Busto F De Arriaga D Ferrero MA Rodriguez-Aparicio LB Martinez-Blanco H Reglero A A normalized plot as a novel and time-saving tool in complex enzyme kinetic analysis Biochem J 2001 358 573 583 11577687 10.1042/bj3580573 PMC1222113

[B37] Mannervik B Regression analysis, experimental error, and statistical criteria in the design and analysis of experiments for discrimination between rival kinetic models Methods Enzymol 1982 87 370 390 7176922 10.1016/s0076-6879(82)87023-7

[B38] Mushtaq A Payton M Sim E The COOH terminus of arylamine N-acetyltransferase from Salmonella typhimurium controls enzymic activity J Biol Chem 2002 277 12175 12181 10.1074/jbc.M104365200 11799105

[B39] Sandy J Holton S Fullam E Sim E Noble M Binding of the anti-tubercular drug isoniazid to the arylamine N-acetyltransferase protein from Mycobacterium smegmatis Protein Sci 2005 14 775 782 10.1110/ps.041163505 15722451 PMC2279269

[B40] Delgoda R Lian LY Sandy J Sim E NMR investigation of the catalytic mechanism of arylamine N-acetyltransferase from Salmonella typhimurium Biochim Biophys Acta 2003 1620 8 14 12595067 10.1016/s0304-4165(02)00500-7

[B41] Delgoda R A Study of Arylamine N-acetyltransferase from Salmonella typhimurium D Phil Thesis 1999 Oxford, University of Oxford

[B42] Westwood IM Kawamura A Fullam E Russell AJ Davies SG Sim E Structure and mechanism of arylamine N-acetyltransferases Curr Top Med Chem 2006 6 1641 1654 10.2174/156802606778108979 16918475

[B43] Expasy ProtParam tool http://us.expasy.org/tools/protparam.html

[B44] Gasteiger E Hoogland C Gattiker A Duvaud A Wilkins M Appel RD Bairoch A Walker JM Protein Identification and Analysis Tools on the ExPASy Server The Proteomics Protocols Handbook 2005 Totowa, Humana Press 571 607

[B45] KyPlot v2.0 beta 13 http://www.woundedmoon.org/win32/kyplot.html

[B46] Guex N Peitsch MC SWISS-MODEL and the Swiss-PdbViewer: an environment for comparative protein modeling Electrophoresis 1997 18 2714 2723 10.1002/elps.1150181505 9504803

