# Supplementary material for: Kinetic characterisation of arylamine N-acetyltransferase from Pseudomonas aeruginosa
Source: BMC Biochem. 2007 Mar 20;8:3. doi: 10.1186/1471-2091-8-3 (PMC1851014; doi:10.1186/1471-2091-8-3)
Supplement: Additional file 1 — Flow diagram describing the normalised plot method. A flow diagram outlining how data from the normalised plot method are analysed. [file 1471-2091-8-3-S1.doc]

**Additional file 1**. Flow diagram describing the normalised plot method. Assays were designed as described in the text. Normalised rates and concentrations are defined in equations 1 and 2. The *F*-test described by Mannervik was used to compare the kinetic models [37].

**Determine Initial Rates**

**in series: *a* = 1, *b* = 1 and *a* = *b***

**Derive a normalised rate equation to allow for all physically possible mechanisms**

**Fit normalised rates to normalised rate equation by simultaneous least squares non-linear regression for all data**

**Are all parameters physically meaningful?**

**YES**

**NO**

**Revise equation**

**Compare kinetic models through goodness of fit and similarity of calculated and estimated values for *den***

**Determine significance of kinetic model election by *F*-statistic comparisons**

**Normalise rates**
