# Supplementary material for: Kinetic characterisation of arylamine N-acetyltransferase from Pseudomonas aeruginosa
Source: BMC Biochem. 2007 Mar 20;8:3. doi: 10.1186/1471-2091-8-3 (PMC1851014; doi:10.1186/1471-2091-8-3)
Supplement: Additional file 2 — Hanes plot for AcCoA as a substrate of PANAT with the acetyl acceptor p-anisidine. This Hanes plot shows the determination of the apparent Michaelis constant for acetyl coenzyme A with the PANAT enzyme in the presence of the acceptor substrate p-anisidine. [file 1471-2091-8-3-S2.doc]

**Additional file 2**. Hanes plot for AcCoA as a substrate of PANAT with the acetyl acceptor *p*-anisidine. The *y*-intercept gives *K*_m,app_/*V*_max,app_, the *x*-intercept gives -*K*_m,app_ for AcCoA and the gradient gives 1/*V*_max,app_ (= 1/(*k*_cat,app_ × [E]_t_) where [E]_t_ is the total concentration of enzyme). Each point marks the mean ± S.D. from duplicate determinations, and the errors have not been weighted. The *y*-coordinate has dimensions of 1 × 10^3^ min and the *x*-coordinate has dimensions of µM. The concentration of *p*-anisidine was 2 mM.
